# Supplementary figures and images for: Real-time resolution studies of the regulation of pyruvate-dependent lactate metabolism by hexokinases in single cells
Source: PLoS One. 2023 Nov 2;18(11):e0286660. doi: 10.1371/journal.pone.0286660 (PMC10621844; doi:10.1371/journal.pone.0286660)

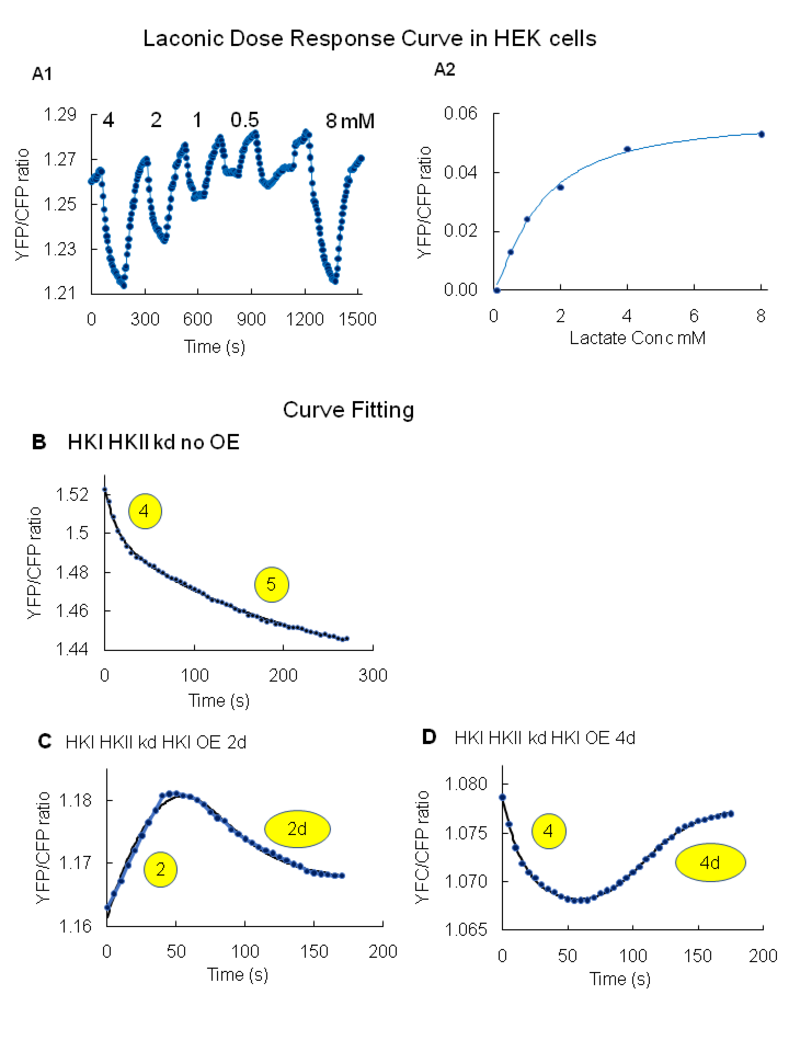

Supplement: S1 Fig — Panels A1 and A2 illustrate a dose response using the FRET sensor Laconic expressed in HEK cells. The cells were exposed to increasing concentrations of lactate as shown in panels A1. The change in FRET ratio was then plotted as a function of concentration and fitted with a Hill equation. The fits yield a Kd of 1.26 mM. This value obtained in intracellular milieu is close to that reported by San Martin et al. in 2013 for in vitro experiments (830±160 μM). Because the FRET ratio values reported in our results are expressed as a percentage of the maximal change obtained at saturating concentration of substrate, these values may be used to get an estimated of the changes in concentration observed under the various experimental conditions. Panels B, C, and D are curve fittings of changes in FRET ratio. In this figure and all the other figures a downward trend in FRET ratio indicates an increase in intracellular lactate. Panel B the increase in intracellular lactate levels evoked by NaCN “phases 4 and 5” were fitted with a sum of two exponential functions. The amplitudes and time constants of the two phases were derived from the fit. In C and D the changes evoked by NaCN with HK overexpression were best fitted by a combination of an exponential and sigmoidal functions. In this case the effects of NaCN on the amplitude of phases 2 and 4 were estimated as the difference between the beginning and end of the fitted traces. When the value at the end of the trace exceeded that at the beginning a negative value for the amplitude was derived from the calculation. (TIF) [file pone.0286660.s001.tif]

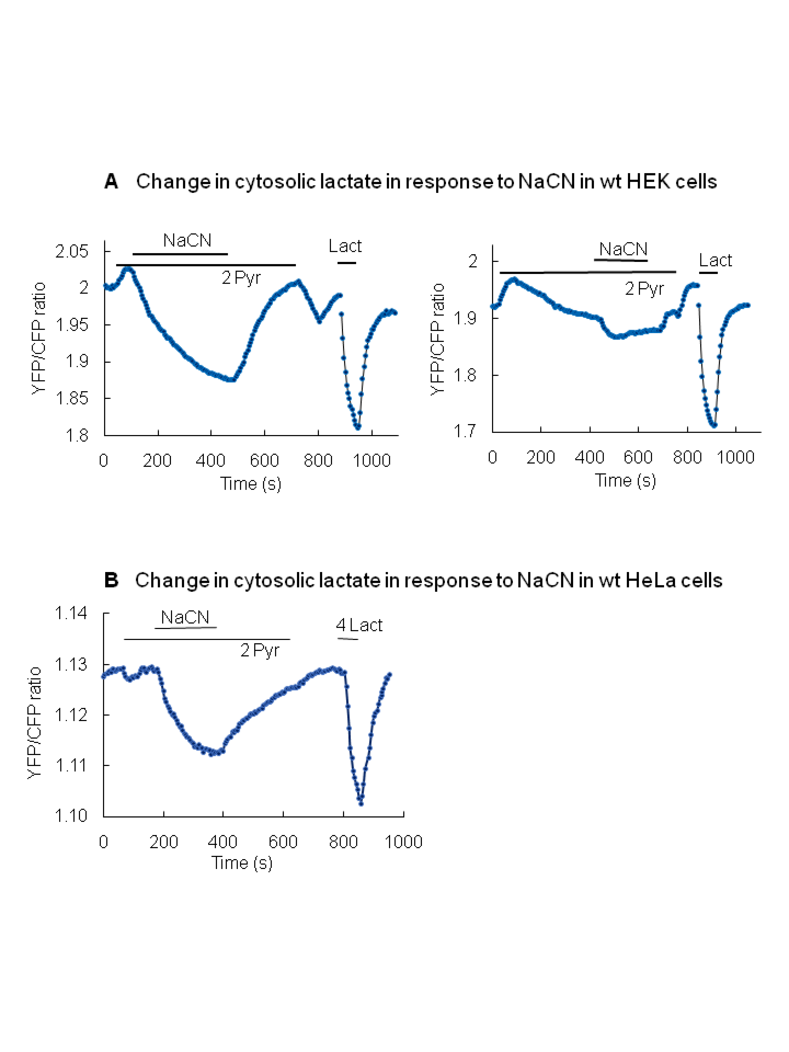

Supplement: S2 Fig — Changes in intracellular concentration of lactate measured using the FRET sensor laconic expressed in wild-type HEK cells. A decrease in FRET ratio reflects an increase in intracellular lactate. These 2 panels illustrate very different response to 2 mM NaCN in 2 adjacent cells. In the left panel addition of pyruvate caused a decrease in lactate and the addition of 2 mM NaCN resulted in a pronounced increase in intracellular lactate level. In contrast the trace in the right panel show that addition of pyruvate caused a transient decrease in lactate and addition of NaCN had little effect in this condition. Panel B. Changes in intracellular concentration of lactate measured using the FRET sensor laconic expressed in wild-type HeLa cells. In HeLa cells as in HEK cells (upper left panel) addition of pyruvate caused a decrease in lactate and the addition of 2 mM NaCN resulted in a pronounced increase in intracellular lactate level. This data validates our measurements made in HEK cells. (TIF) [file pone.0286660.s002.tif]

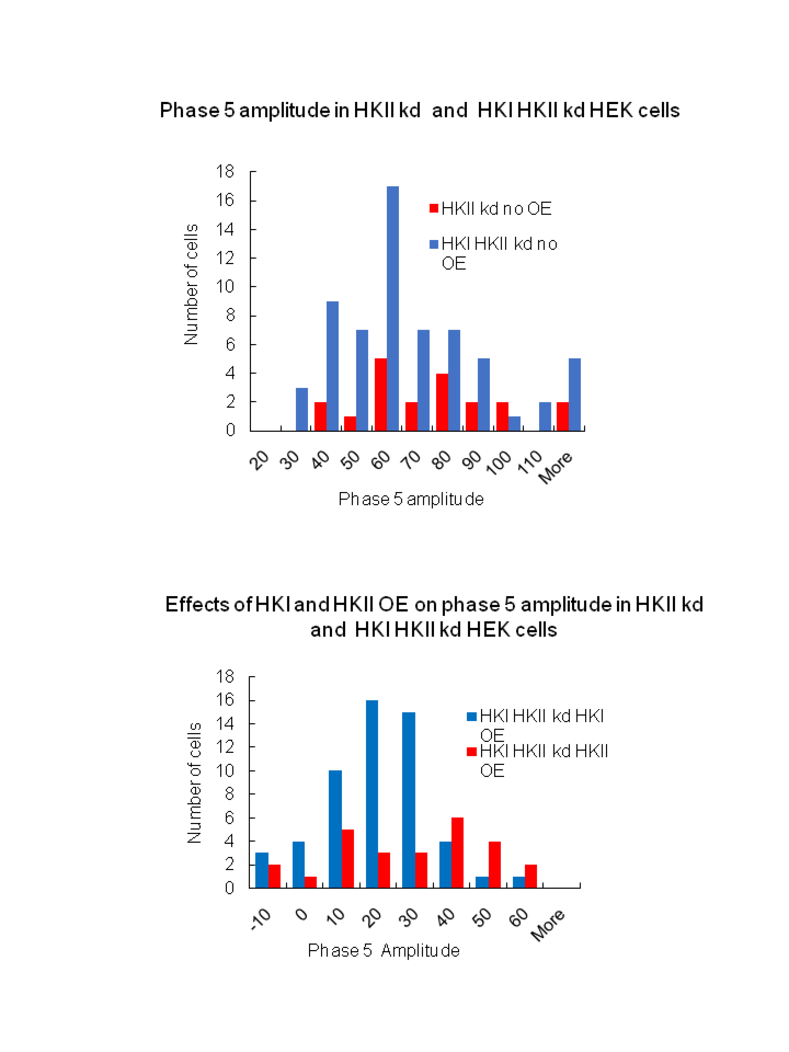

Supplement: S3 Fig — Knocking down only HKII or HKII plus HKI has similar effect on lactate accumulation as a result of mitochondrial inhibition by NaCN. In these experiments there was no further expression (no OE) of HKI or HKII following DOX-induced knock down. For the HKI HKII KD (no OE) the mean normalized amplitude for phase 5 was 64.4 ± 3.55 (n = 63). For HKII KD (no OE) the mean phase 5 normalized amplitude was 71.6 ± 5.08 (n = 21). F-test yielded a P value of 0.12. The t-test (equal variance) P value was 0.97. The shapiro-wilk test P value for HKI HKII KD no OE was 0.1, and 0.07 for HKII. These results support the hypothesis that HKII is the main hexokinase expressed in wild type HEK cells. Lower panel: Overexpression of HKI or HKII has similar effects on lactate accumulation as a result of mitochondrial inhibition by NaCN. These histograms depict the effects of the overexpression (OE) of HKI and of HKII in HEK cells in which HKI and HKII had been previously knocked down. For HKI OE in HEK cells in which HKI HKII had been previously knocked down (KD) the mean normalized amplitude for phase 5 was 16±1.8 (n = 55). For HKII OE in HKI HKII KD HEK cells the mean normalized amplitude for phase 5 was 23.4±3.8 (n = 27). An F-test yielded a P value of 0.012 and a t-test (unequal variance) yielded P<0.0005. The shapiro-wilk test P value for HKI OE was 0.07, and 0.9 for HKII OE. These results strongly suggest that overexpression of HKI or HKII has similar effects on the regulation of lactate metabolism by mitochondria. (TIF) [file pone.0286660.s003.tif]

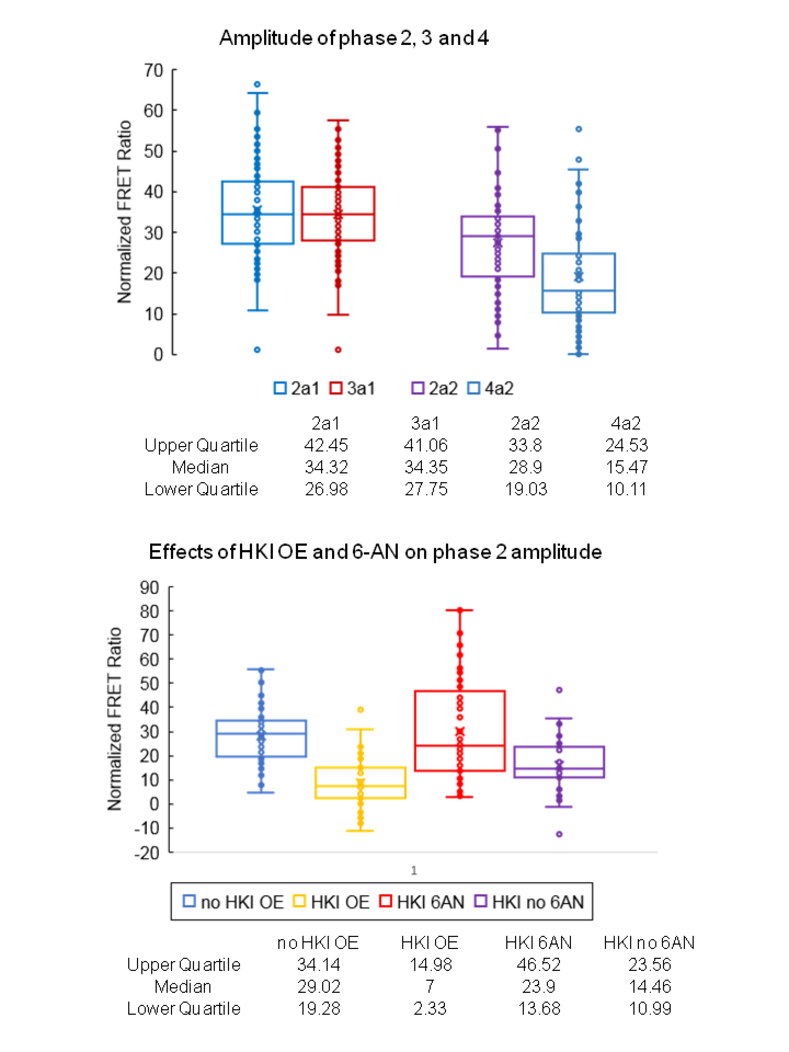

Supplement: S4 Fig — Box-and-whisker plots for the amplitude of phase 2, 3 and 4. It should noted that the 2 plots on the left (2a1, 3a1) and the 2 plots on the right (2a2, 4a2) show the results of two separate sets of experiments in which AOA (2a1, 3a1) and NaCN (2a2, 4a2) were tested. The values shown in the table below the graph were derived from the data used to generate the above plots. Lower graph, box-and-whisker plots for the amplitude of phase 2 without and with overexpression of HKI, and with and without 6-AN in the presence of HKI overexpression. The values shown in the table below the graph were derived from the data used to generate the above plots. The horizontal lines mark the median, the box limits indicate the 25th and 75th percentiles. Outlier points are included. It is to be noted that we report some variability in responses of HEK cells. These cells are clonal and this analysis indicates that even using such “identical” cells variance can be seen. (TIF) [file pone.0286660.s004.tif]

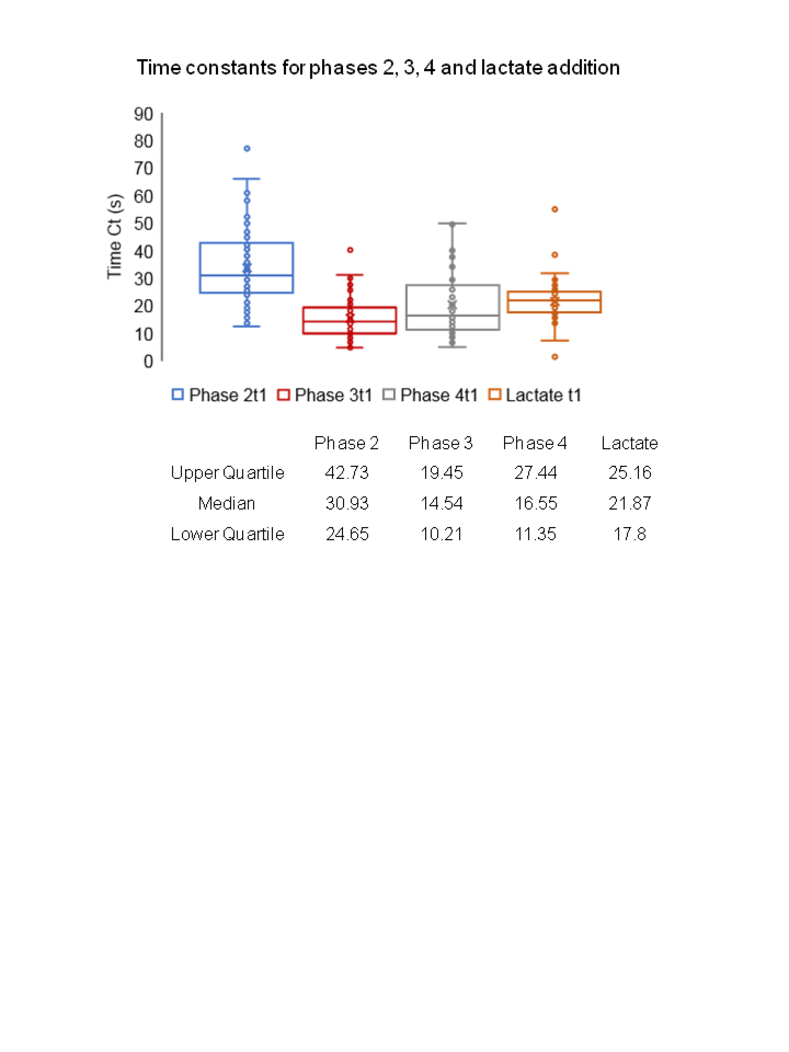

Supplement: S5 Fig — the horizontal lines mark the median, the box limits indicate the 25th and 75th percentiles. The values shown in the table below the graph were derived from the data used to generate the above plots. (TIF) [file pone.0286660.s005.tif]

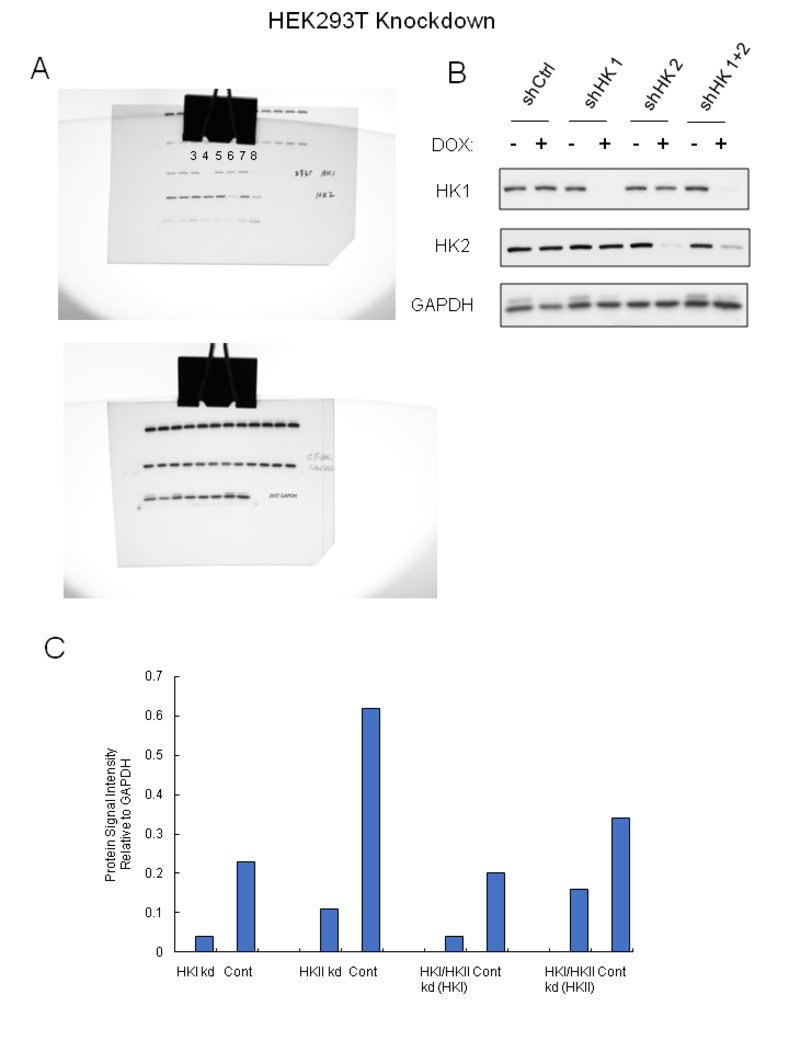

Supplement: S6 Fig — Uncropped gels representing HKI, HKII and HKI/HKII knocked down in HEK cells. Columns 3, 5 and 8 correspond to control conditions without doxycycline. Column 2 is for HKI KD, column 6 for HKII KD and column 8 for the double HKI/HKII KD. Refer also to Fig 1 Panel C for lane labeling. Lower panel: Gel analysis for the 3 conditions depicted above (HKI KD, HKII KD and HKI/HKII KD). The graph was generated using the lane profile function of Image J. Control values (no doxycycline) are shown to the right for each condition. (TIF) [file pone.0286660.s006.tif]
