# Supplementary material for: Real-time resolution studies of the regulation of pyruvate-dependent lactate metabolism by hexokinases in single cells
Source: PLoS One. 2023 Nov 2;18(11):e0286660. doi: 10.1371/journal.pone.0286660 (PMC10621844; doi:10.1371/journal.pone.0286660)
Supplement: Attachment — Submitted filename: Response to reviewers.docx [file pone.0286660.s013.docx]

Response to reviewers:

The authors wish to thank the reviewers for their constructive concerns and comments. We have addressed these comments, and our responses are included in the letter below and in the revised manuscript (yellow highlights).

General comments: We have carried out a number of additional experiments in other cell types e.g. HeLa and IPS cells to validate our studies in HEK cells. Unfortunately, due to the loss of our knockout HEK cell lines – a failure of our liquid nitrogen storage facility- we do not have access to these cells for further experimentation at this time. It had taken us a long time to generate these cell lines and we are trying to get back some of these reagents, but it has been a very slow process.

Specific comments:

Reviewer #1 concerns:
-All experiments were conducted in HEK cells. Key experiments must be shown in at least one additional system.
New data obtained with HeLa cells are presented in Fig S2. This data shows that the effects of pyruvate and NaCN are similar to those observed in HEK cells. We had previously carried out experiments in IPS cells. The FRET studies utilizing the Laconic probe carried out by B.R. in IPS cells showed that inhibition of mitochondrial metabolism varied as a function of glucose phosphorylation (Nakano et al., 2017). Together these data strengthen our studies carried out in HEK cells.

To test for the effects of inhibitors of the glucose 6 phosphate dehydrogenase (G6PDH) or of the malate aspartate shuttle (MAS) we would have to knock down HKI and HKII in a cell line such as HeLa cells. This would require several months of work for us. Our goal is indeed to demonstrate the validity of our hypotheses. But to this end we propose to knock down Aspartate aminotransferase (GOT1) to further establish the role of MAS in pyruvate-induced lactate metabolism, and knock down G6PDH to establish the role of HKs in regulating this process. These enzymes have already been knocked down in HEK cells and we hope to be able to collaborate with the laboratories that generated these cells lines once our work is published.

Our ultimate goal is to carry out these studies in adult cardiac myocytes (ARVM) and neonatal cardiac myocyte (NRVM), which have very different metabolism, different HKs content and different resistance to ischemia.

-Representative images of the FRET must be shown in main or supplementary figures to illustrate robustness of signal and system in additional to only the quantified data.
We have amended Fig 1 to show images of the change in FRET ratio evoked by the addition of lactate to the media and recorded simultaneously from individual HEK cells. These images underlie the strength of the FRET signals and their reproducibility in uniform response- rates and amplitude. They are but an illustrative example of the approach we have adopted throughout the experimental paradigms investigated.

- Figure 1a. Dox label is incomplete, and western bands are not aligned in different panels.
Amended accordingly. We have also added uncropped image of the gels under supporting information (Fig S6).

- Many figures do not indicate number of experimental repeats, and need to have statistics and error bars shown (e.g. 4b-d)
We have added a number of graphs with error bars and all the relevant information in Fig 2C, Fig 5A&B, Fig S4 and S5. The latter two figures are box plots showing the individual data points.

Reviewer #2:  Recommendation –
1. Can the authors elaborate on how they are achieving single cell resolution in the FRET sensor assays with laconic? I understand that genetically-encoded reporters will give a higher resolution for the FRET ratios but is it really done at a single cell level? Are there cell sorting or isolation methods which are being used to resolve to single cell basis? I read through the FRET sensor and imaging method space but is not immediately clear. If it is transfected cDNAs in HEK cells then the assay is more heterogenous if the cells are not manipulated/sorted post transfection. Maybe I am missing something here and need more clarification how it is single cell resolution?
Indeed a couple of our colleagues who initially reviewed our manuscript made the comment that we should further detail the FRET technique that we use. We have inserted images in Fig 1 to better explain our approach. This is also further detailed in “Methods”. Yes, the imaging experiments are carried out on single cells cultured on microscope cover slips. We image between 3 to 6 cells at a time and recordings are done from individual ROI. We make recordings from 30 to 100 cells for each experimental condition. We hope that this addendum will address Reviewer #2 comment.

2. Just a bit of image alignment here but the labels on Fig1A for -/+ dox are shifted and not aligning with the lanes on the images I received. A graphical normalization of knockdown can also be presented in the supplemental figures.
Amended accordingly see also Fig S6.for graphical analysis.

3. Have the authors investigated ROS activity in the HKI and HKII knockdown cells? This might be useful to related to how lactate utilization can be affect by ROS and also even in states of increased ROS activity if the MA shuttle becomes further de-regulated by HK inhibition along with increase in ROS.
Thank you for the insightful comment. Indeed, we do have a ROS sensitive fluorescent probe and we had carried out some experiments with it a couple of years ago, but we did not pursue this line of research after we lost our HKI/HKII knockdown cells. Our goal is still to further investigate the role of MAS in the regulation of the metabolism of lactate, but our plan now will be to directly target GOT1. Please see also our answer to reviewer #1 comment #1 and our general comment..

Thank you,

Bernard Ribalet for the authors
