# Supplementary material for: Real-time resolution studies of the regulation of pyruvate-dependent lactate metabolism by hexokinases in single cells
Source: PLoS One. 2023 Nov 2;18(11):e0286660. doi: 10.1371/journal.pone.0286660 (PMC10621844; doi:10.1371/journal.pone.0286660)
Supplement: Attachment — Submitted filename: Response to reviewers 3 4 2023.docx [file pone.0286660.s014.docx]

Los Angeles 3-4-2023

Response to the Reviewers comments.

***Reviewer #2: Thank you for your response. I truly believe utilizing HK1 and HKII knockdown cells would be a good line of questioning besides the GOT1 line you have mentioned. Best of luck on your future research endeavors.***
This sounds positive to us and we thank the reviewer for their review and encouragement..

***Reviewer #3: The authors are presumably performing their studies under atmospheric conditions enriched with CO2, hyperbaric conditions, as compared to in vivo. In light of the normoxic oxygen levels ranging from ~0-13%, it is difficult to relate the lactate utilization to what might be actually occurring. This confounding issue should be addressed.***
This is confusing to us and colleagues in our Department! What does the reviewer mean by hyperbaric conditions, enriched in CO_2_?
First, our experimental conditions are the same as those used by laboratories around the world to study metabolism, or any cellular event for that matter, in cultured cells. These conditions are: room temperature and atmospheric pressure, water saturated, with trace amounts of CO_2_ close to 0.04% and O_2_ close to 21%. We cannot figure out where the enriched CO_2_ and 0-13% O_2_ come from, at no point in the manuscript did we mention the use of hyperbaric conditions. Under our experimental conditions, with 21% O_2_ mitochondrial respiration is very efficient and allows for study of lactate utilization.
If the reviewer is referring to the culture conditions, yes, the CO_2_ level in the incubator is 5% and the O_2_ level is close to 16% (21-5=16%), the standard tissue culture conditions. Furthermore, these are the concentrations found in the lung’s alveoli and are therefore the *in vivo* conditions. Again in this case the pressure inside the incubator is normal.

***It is indeed unfortunate when there is a loss of a critical reagent such as the knockdown HEK cells. We've all been there. However, it's loss and lack of repeat of findings in another cell line wherein the genes were knocked out means that no one else can repeat the study for independent validation.***
Why is it that no one else can repeat our studies for independent validation?

- For one thing, almost every laboratory nowadays can knock down genes in cultured cells.
- Second. if someone is willing to spend $4000, the HEK hexokinase knockdown cells are commercially available.
   [*https://www.abcam.com/human-hk2-hexokinase-ii-knockout-hek-293-cell-line-ab269485.html*](https://www.abcam.com/human-hk2-hexokinase-ii-knockout-hek-293-cell-line-ab269485.html)
- And third, as our recent data show, it is possible to replicate the experimental data obtained with hexokinase knockdown cells, by simply removing glucose form the perfusion medium. A predicted result since we have demonstrated that the effect of hexokinase requires the enzyme to be catalytically active and glucose is the only substrate for hexokinase.

Any interested laboratory can use one or several of these approaches to replicate our findings.

***Nor has the requirement for the findings to be replicated in another cell line been met.***
This is simply not true. We may not have knockdown hexokinase in another cell line, but we have replicated most of the data presented in the submitted manuscript in a number of cell systems.

- First, we added in supplemental data experimental results that were obtained in Hela cells. These results show that the responses to pyruvate and NaCN, in the absence of glucose, are almost identical to the response obtained in HEK cells wild type and hexokinase knockdown.
- We also quoted in the manuscript that similar results were obtained in IPS cells exposed to solutions with and without glucose.
- In addition, similar responses to pyruvate in the presence and absence of glucose were obtained in neonatal rat ventricular myocytes (NRVM).

All of this indicates that what we observe in HEK cells are properties that are common to a variety of cell types, primary and immortalized.

It is true that we have not knocked down hexokinase in a cell line other than HEK cells, but as we have already outlined, an alternative approach consisting of removing glucose with Hela cells achieves similar results. These observations demonstrate that the changes in lactate metabolism observed in wild type and manipulated HEK cells are common to other cell types.

In view of these observations, knocking down hexokinase in another cell line would not significantly strengthen our hypothesis. Instead, we are aiming at knocking down enzymes of the malate aspartate shuttle (GOT1) and pyruvate carboxylase to validate our model for pyruvate-induced lactate utilization.

***The graphs are still in need of being cleaned up with proper spacing in titles and “R” values need to be readable.***
We can space the “R” values so that they are easy to read.

In summary, our experimental work and manuscript preparation indicates the thoroughness and reproducibility of the hypothesis. The comments from Reviewer # 3 are unwarranted especially given the misconceptions regarding our experimental conditions (O_2_ and CO_2_ levels), and the lack of reproducibility for data validation. On this basis we respectfully request that the Editor reconsider his decision to decline publication of our work.

Sincerely,

Bernard Ribalet
For the authors
