# Supplementary material for: Real-time resolution studies of the regulation of pyruvate-dependent lactate metabolism by hexokinases in single cells
Source: PLoS One. 2023 Nov 2;18(11):e0286660. doi: 10.1371/journal.pone.0286660 (PMC10621844; doi:10.1371/journal.pone.0286660)
Supplement: Attachment — Submitted filename: Reviewer#4_2023.docx [file pone.0286660.s015.docx]

Los Angeles 1-27-2023

Thank you to Reviewer #4 for the insightful comments.

Answers to these specific comments:

Question #1. How might glutamate (and/or malate) compare to pyruvate with respect to lactate removal? In other words, is the pyruvate-dependent lactate oxidation observed in the study a unique function of pyruvate, or can another substrate accomplish the same? Such a comparison would potentially tease out whether PDH activation vs MAS activation are responsible for the pyruvate-dependent decline in lactate observed in the study.

Answer #1. We have spent a fair amount of time looking at mitochondrial substrates that may mimic the effect of pyruvate in lowering intracellular lactate, paying particular attention to malate and glutamate. As shown in the traces below, both substrates increase intracellular lactate. The effect of malate is fast and small, while that of glutamate is more substantial, slow acting and poorly reversible (for these experiments the cells were cultured overnight in the absence of glutamine). We investigated the effect of malate in the presence and absence of HKI and saw no major differences. These initial studies enabled us to refine our approach and report the more substantive results and interpretation in the current submission.

|  |  |
| --- | --- |
| 2.54 | 1.18861 |
| 7.54 | 1.18818 |
| 12.55 | 1.18833 |
| 17.55 | 1.18815 |
| 22.55 | 1.18868 |
| 27.55 | 1.18816 |
| 32.55 | 1.18847 |
| 37.55 | 1.18867 |
| 42.55 | 1.18813 |
| 47.55 | 1.18691 |
| 52.55 | 1.18575 |
| 57.55 | 1.18504 |
| 62.55 | 1.18434 |
| 67.55 | 1.18445 |
| 72.55 | 1.18451 |
| 77.55 | 1.18442 |
| 82.55 | 1.18468 |
| 87.55 | 1.18488 |
| 92.55 | 1.1852 |
| 97.55 | 1.18539 |
| 102.55 | 1.18529 |
| 107.55 | 1.18559 |
| 112.55 | 1.18568 |
| 117.55 | 1.18626 |
| 122.55 | 1.18737 |
| 127.55 | 1.18855 |
| 132.54 | 1.18938 |
| 137.54 | 1.18962 |
| 142.54 | 1.18985 |
| 147.54 | 1.18993 |
| 152.54 | 1.18999 |
| 157.54 | 1.18983 |
| 162.54 | 1.18976 |
| 167.54 | 1.18986 |
| 172.54 | 1.18975 |
| 177.54 | 1.18951 |
| 182.54 | 1.18905 |
| 187.54 | 1.18765 |
| 192.54 | 1.18653 |
| 197.54 | 1.18566 |
| 202.54 | 1.18542 |
| 207.54 | 1.18557 |
| 212.54 | 1.18528 |
| 217.54 | 1.1856 |
| 222.54 | 1.18588 |
| 227.54 | 1.18611 |
| 232.54 | 1.18631 |
| 237.54 | 1.18658 |
| 242.54 | 1.18691 |
| 247.54 | 1.18695 |
| 252.54 | 1.18757 |
| 257.55 | 1.18859 |
| 262.55 | 1.1895 |
| 267.55 | 1.18972 |
| 272.55 | 1.18991 |
| 277.55 | 1.19023 |
| 282.55 | 1.1901 |
| 287.55 | 1.18984 |
| 292.55 | 1.18977 |
| 297.55 | 1.18968 |
| 302.55 | 1.18929 |
| 307.55 | 1.18922 |
| 312.55 | 1.18911 |
| 317.55 | 1.18908 |
| 322.55 | 1.18892 |
| 327.55 | 1.18833 |
| 337.55 | 1.18044 |
| 342.55 | 1.17048 |
| 347.55 | 1.15296 |
| 352.55 | 1.14065 |
| 357.55 | 1.13271 |
| 362.55 | 1.12727 |
| 367.55 | 1.12348 |
| 372.55 | 1.12059 |
| 377.55 | 1.11818 |
| 382.55 | 1.11626 |
| 387.55 | 1.11471 |
| 392.55 | 1.11327 |
| 397.55 | 1.11207 |
| 402.55 | 1.11086 |
| 407.55 | 1.10981 |
| 412.55 | 1.10906 |
| 417.55 | 1.1084 |
| 422.55 | 1.10757 |
| 427.55 | 1.10666 |
| 432.55 | 1.10618 |
| 437.55 | 1.11034 |
| 442.55 | 1.11909 |
| 447.55 | 1.12903 |
| 452.55 | 1.13728 |
| 457.55 | 1.14502 |
| 462.55 | 1.15075 |
| 467.55 | 1.1551 |
| 472.55 | 1.1585 |
| 477.55 | 1.16116 |
| 482.55 | 1.16314 |
| 487.55 | 1.16482 |
| 492.55 | 1.16632 |
| 497.55 | 1.16773 |
| 502.55 | 1.16852 |
| 507.55 | 1.16978 |
| 512.55 | 1.1707 |
| 517.55 | 1.17141 |
| 522.55 | 1.17212 |
| 527.55 | 1.17297 |
| 532.55 | 1.17377 |

|  |  |
| --- | --- |
| 2.03 | 1.08392 |
| 7.03 | 1.0836 |
| 12.03 | 1.0834 |
| 17.03 | 1.08294 |
| 22.03 | 1.0826 |
| 27.03 | 1.08207 |
| 32.03 | 1.0831 |
| 37.03 | 1.08395 |
| 42.03 | 1.08364 |
| 47.03 | 1.08348 |
| 52.03 | 1.08314 |
| 57.03 | 1.08243 |
| 62.03 | 1.08172 |
| 67.03 | 1.08093 |
| 72.03 | 1.07991 |
| 77.03 | 1.07908 |
| 82.03 | 1.07824 |
| 87.03 | 1.07746 |
| 92.03 | 1.07634 |
| 97.03 | 1.07589 |
| 102.03 | 1.07494 |
| 107.03 | 1.07408 |
| 112.03 | 1.07349 |
| 117.03 | 1.07284 |
| 122.03 | 1.07207 |
| 127.03 | 1.0715 |
| 132.03 | 1.07065 |
| 137.03 | 1.07016 |
| 142.03 | 1.0695 |
| 147.03 | 1.06912 |
| 152.03 | 1.06853 |
|  | 1.06785 |
| 157.03 | 1.0673 |
| 162.03 | 1.06695 |
| 167.03 | 1.06622 |
| 172.03 | 1.06575 |
| 177.03 | 1.06511 |
| 182.03 | 1.06479 |
| 192.03 | 1.0622 |
| 197.03 | 1.06053 |
| 202.03 | 1.0598 |
| 207.03 | 1.05879 |
|  | 1.05891 |
| 212.03 | 1.05885 |
| 217.03 | 1.05883 |
| 222.03 | 1.05896 |
| 227.03 | 1.05906 |
| 232.03 | 1.05941 |
| 237.03 | 1.05938 |
| 242.03 | 1.05974 |
| 247.03 | 1.05972 |
| 252.03 | 1.0599 |
| 257.03 | 1.05992 |
| 262.03 | 1.06008 |
| 267.03 | 1.06024 |
| 272.03 | 1.06039 |
| 277.03 | 1.06045 |
|  | 1.06065 |
| 282.03 | 1.06065 |
| 287.03 | 1.0608 |
| 292.03 | 1.06071 |
| 297.03 | 1.06054 |
| 302.03 | 1.06065 |
| 312.03 | 1.05954 |
| 317.03 | 1.05871 |
| 322.03 | 1.05898 |
| 327.03 | 1.05915 |
| 332.03 | 1.05933 |
| 337.03 | 1.05868 |
| 342.03 | 1.05853 |
| 347.03 | 1.05827 |
|  | 1.05764 |
| 357.03 | 1.05044 |
| 362.03 | 1.03936 |
| 367.03 | 1.03094 |
| 372.03 | 1.02554 |
| 377.03 | 1.02228 |
| 382.03 | 1.02032 |
| 387.03 | 1.01906 |
| 392.03 | 1.0183 |
| 397.03 | 1.01757 |
| 402.03 | 1.01715 |
| 407.03 | 1.01706 |
| 412.03 | 1.01672 |
| 417.03 | 1.01676 |
| 422.03 | 1.01626 |
|  | 1.01792 |
| 432.03 | 1.02386 |
| 437.03 | 1.03129 |
| 442.03 | 1.03834 |
| 447.03 | 1.04313 |
| 452.03 | 1.04655 |
| 457.03 | 1.04864 |
| 462.03 | 1.05055 |
| 467.03 | 1.05211 |
| 472.03 | 1.05327 |
| 477.03 | 1.05385 |
| 482.03 | 1.05428 |

Question #2. In this study, pyruvate carboxylase is considered and tested. How might malic enzyme contribute to the proposed model?

Answer #2. This is an interesting question. We have studied the role of the malic enzyme ME1, using the inhibitor ME1*. The results of these experiments were reported in an earlier manuscript that was rejected due in part to its complexity. These experiments showed that addition of ME1* blocks pyruvate-induced lactate oxidation as well as NaCN-induced lactate accumulation. The latter observation will be included in a second manuscript to be submitted upon successful publication of the data within the current manuscript. We have now included, in the present manuscript, the effect of ME1* on pyruvate-evoked decrease in intracellular lactate to address the Reviewer’s comment (see fig 5) Based on these results we postulate that pyruvate carboxylation via ME1 may account for the activation of the TCA cycle and the transformation of lactate into pyruvate in the intermembrane space (fig 4 in the manuscript).

This alone cannot account for pyruvate-evoked lactate depletion since malate does not have the same effect (see above). One possible explanation to account for this discrepancy may be that NAD(P) produced during the transformation of pyruvate into malate is essential for the activation of the malate aspartate shuttle and the transformation of lactate into pyruvate.

To best address this question we will have to knock down ME1, to help determine how pyruvate and NaCN modulate intracellular lactate levels in our system.

Sincerely,

Bernard Ribalet
